# Trauma-induced exostosis of multiple suture lines causing partial bilateral nasolacrimal duct obstruction in a 7-year-old Thoroughbred mare

**DOI:** 10.4102/jsava.v90i0.1764

**Published:** 2019-09-26

**Authors:** Luke A. Poore, Christelle le Roux, Ann Carstens

**Affiliations:** 1 Department of Companion Animal Clinical Studies, Faculty of Veterinary Science, University of Pretoria, Pretoria, South Africa

**Keywords:** horse, trauma, computed tomography, exostosis, suture lines

## Abstract

A definitive diagnosis of extensive suture line exostoses affecting the nasofrontal, nasolacrimal, nasomaxillary, frontolacrimal, lacrimozygomatic and lacrimomaxillary suture lines in a 7-year-old Thoroughbred mare with chronic bilateral epiphora and facial deformation was achieved using standing computed tomography (CT) examinations. Positive contrast dacryocystorhinography using CT revealed partial bilateral obstruction of the nasolacrimal ducts. Minimally displaced depression fractures of the right nasal bone, the right maxillary bone and right frontal bone were also demonstrated. The cosmetic appearance of the periosteal reaction associated with the suture line exostosis and epiphora significantly improved within 3 months of diagnosis and treatment.

## Introduction

**FIGURE 1:**
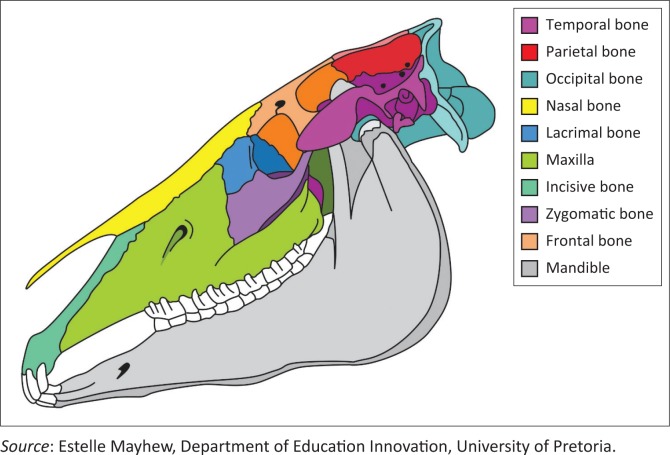
Diagrammatic representation of the equine cranium from the lateral perspective illustrating the craniofacial suture lines.

**FIGURE 2:**
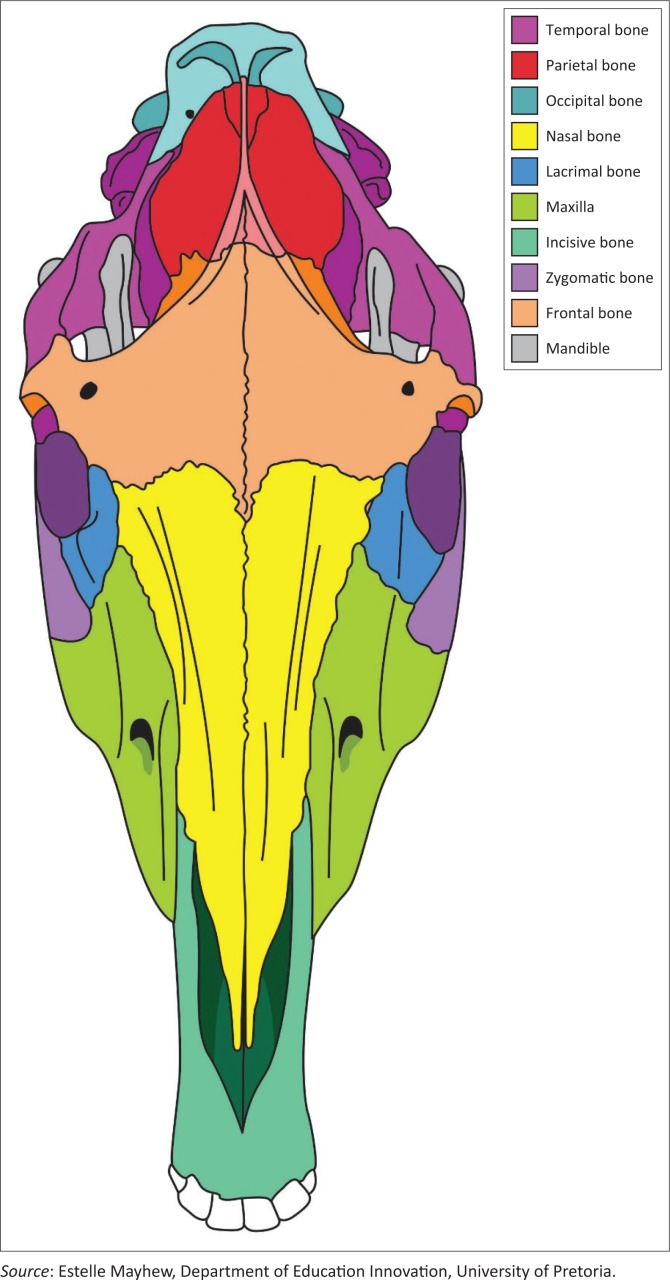
A diagram of the equine cranium from the dorsal perspective illustrating the craniofacial suture lines.

The equine skull is composed of many flat bones bound together by collagen fibres and overlying periosteum (Dixon 2014; Klein et al. 2014; Smallwood et al. 2002; Williams & Warwick 1980). Craniofacial sutures are fibrous joints present in the skull and the nasofrontoal, nasolacrimal, nasomaxillary and frontolacrimal especially are of clinical importance (Figures 1 and 2). These suture lines are the primary sites of equine craniofacial bone growth in the horse and fuse in the first few months of life, becoming ossified between 3 months and 5 years of age (Bonilla, Wilson & Sangschi 2015; Butler et al. 2009). These suture lines allow small movements before they close, which absorb mechanical stress, distribute masticatory forces, allow passage through the birth canal and contribute to the elastic properties of the skull (Dixon 2014; Rafferty & Herring 1999; Rafferty, Herring & Marshall 2003; Rice 2008).

Inflammation and suture line exostosis of the equine craniofacial suture lines result in regional swellings and is a common cause of firm facial swellings in the horse (Carslake 2009; Dixon 1991, 2014; Klein et al. 2014; Lane et al. 1987; Manso-Diaz & Taeymans 2012; Tremaine & Dixon 2001a). Suture exostoses are normally areas of non-painful bone proliferation that usually regress with time (Dixon 1991; Gibbs & Lane 1987; Klein et al. 2014; Tremaine & Dixon 2001a). As the nasolacrimal ducts are imbedded in the ventral aspect of the nasal and frontal bones, nasofrontal suture line exostoses with local inflammation and bone proliferation can cause partial or complete obstruction of the nasolacrimal ducts with secondary epiphora (Bonilla et al. 2015; Carslake 2009; Klein et al. 2014; Manso-Diaz & Taeymans 2012).

This case report describes the clinical, radiographic and computed tomographic findings of suture line exostoses of the nasofrontal, nasolacrimal, frontolacrimal, lacromaxillary and lacrimozygomatic craniofacial suture lines in a skeletally mature Thoroughbred mare who suffered severe facial trauma. The use of computed tomography (CT) with dacryocystorhinography has not previously been reported in a standing horse.

### Ethical considerations

Ethical clearance was not needed or required for the study.

## Case report

A 7-year-old Thoroughbred mare was presented to the Onderstepoort Veterinary Academic Hospital (OVAH) for the evaluation of a severely painful facial swelling of approximately 6 weeks’ duration. The owners reported that she had suffered severe violent blunt trauma directly to the facial region that resulted in extensive facial swelling over the following 48-hour period. Intermittent treatment with non-steroidal anti-inflammatory medicines and stable confinement in the 6 weeks prior to referral had failed to resolve the facial swelling, and bilateral epiphora had developed. Additionally, bilateral conjunctivitis developed 5 days prior to presentation at the OVAH.

**FIGURE 3:**
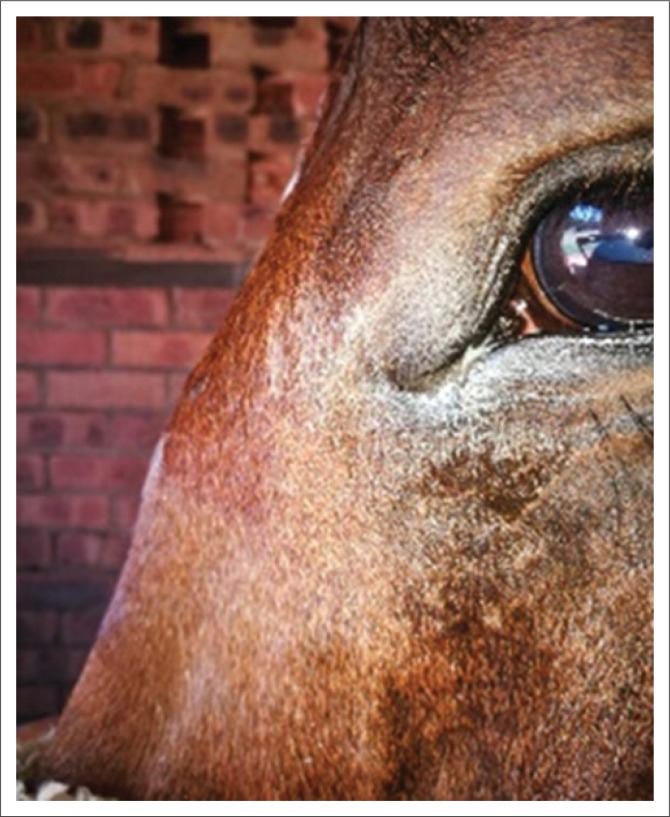
A left-sided photograph of the head at initial presentation showing a firm swelling at the nasofrontal suture line and a firm swelling adjacent to the medial canthus of the left eye consistent with the nasolacrimal suture line.

Clinical examination at presentation showed normal clinical parameters and mentation. Bilateral epiphora with a purulent discharge from the medial canthi and three discrete firm prominent swellings situated between the eyes in the areas of the frontal, nasal and lacrimal bones (Figure 3) were observed. There was a severe pain response evident with palpation of the facial swellings. Routine ocular and regional adnexa examinations using a pen torch, direct ophthalmoscopy, fluorescein staining of the cornea, menace response testing and pupillary light reflex testing were unremarkable apart from bilateral epiphora and conjunctivitis. A Jones test was negative on both eyes.

**FIGURE 4:**
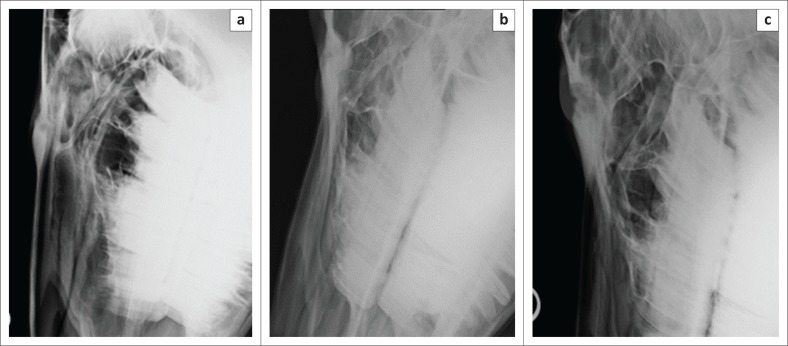
(a) Latero-lateral, (b) LeDRtVO and (c) RtDLeVO radiographic projections of the affected area, demonstrating the smooth solid periosteal reaction associated with an irregular radiolucent fissure line and a poorly marginated oval radiolucency associated with this feature particularly obvious in (c). Also note the depressed appearance of the caudal nasal and rostral frontal bones, especially prominent in (a).

Radiographic and CT examinations with retrograde dacryocystorhinography, under a standing sedation protocol, were performed. An intravenous catheter was aseptically placed in the left jugular vein (Extended Use Milocath, Mila International, Kentucky, United States), and the mare was sedated using intravenous detomidine hydrochloride (13 mg/kg bodyweight [bwt] Domosedan, Pfizer Animal Health, Sandton, South Africa) and intravenous butorphanol tartrate (26 mg/kg bwt Torbugesic, Fort Dodge Animal Health, Midrand, South Africa). Radiographic evaluation of the head was performed using a ceiling-mounted computed radiography unit (Apelem Magnum 80 Medical Technology Italy). Latero-lateral and the oblique (RtDLeVO and LeDRtVO) radiographic projections (Figure 4a, b and c) revealed smooth solid periosteal proliferation with an irregular radiolucent fissure line and a poorly marginated oval radiolucency associated with this, rostral to the orbit in the region of the nasofrontal suture. Focal depression of the caudal aspect of the nasal bone and nasofrontal suture region, with marked sclerosis superimposed over the conchofrontal sinus region, was also noted. The findings were consistent with extensive nasofrontal suture exostosis and a focal healed depression fracture. Computed tomography (CT) examination was performed the following day with a dual slice helical scanner (Siemens Somatom Emotion Duo Siemens Medical Systems, Forchheim, Germany) with a sliding gantry. Images were acquired in a bone window (window level [WL]: 450 Hounsfield units [HU] and window width [WW]: 1500 HU) using 3-mm slice thickness, and 50% reconstruction increments, with windowing and multiplanar image reconstruction performed as needed.

**FIGURE 5:**
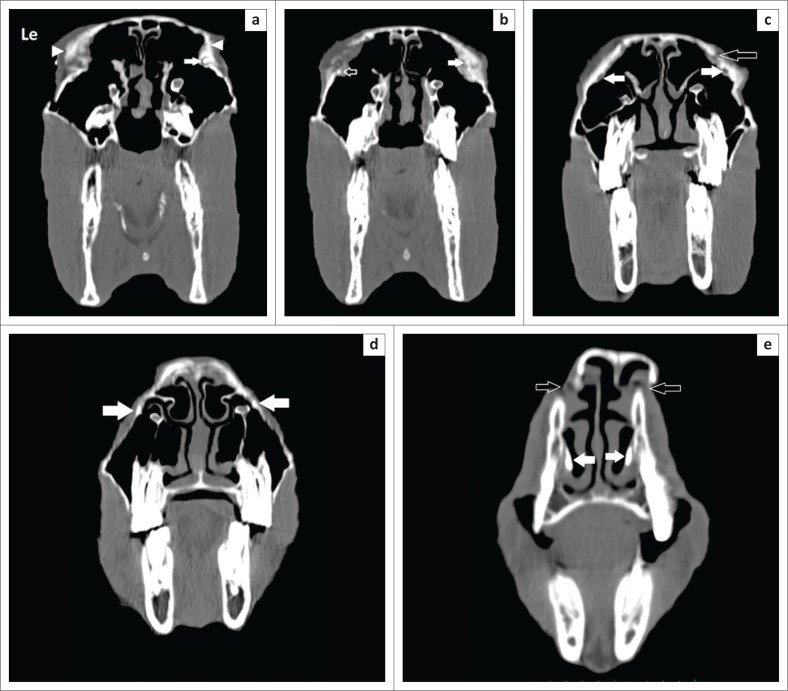
A sequence of slightly oblique transverse computed tomography images are presented from caudal (level of the medial canthus of the eyes) (a) to rostral (e). In (a), the contrast medium is present within the right ventral nasolacrimal puncta (white arrow) but is not visualised on the left. Note the marked bilateral lacrimal bone periosteal proliferation present (white arrowheads). In (b), the contrast material is not clearly visualised on the right (white arrow), in the region of the most severe bony proliferation, but is present on the left as a focal region of hyperattenuation (open arrow). In (c), at the level of the caudal maxillary molars, the contrast material is not visible in either nasolacrimal duct (white arrows), and a focal cortical defect in the lacrimal bone is visualised. In (d), a small amount of contrast attenuation can be visualised within both nasolacrimal ducts (white arrows) with the contrast present in the nasal passages (white arrows) in (e), immediately caudal to the nasal puncta. Note the discontinuity and displacement of the dorsal-most aspect of the nasal bones (open arrows), which is because of patient motion and thus is artefactual. The left (Le) of the patient is on the left of the image, and the dorsal is to the top. Images were obtained under standing sedation, in a bone window (WL: 450 HU and WL: 1500 HU).

Bilateral retrograde dacryocystorhinography was performed using 15 mL of Iohexol (Omnipaque 300 mg I/mL, GE Healthcare, Midrand, South Africa), with a 15-cm 6-FR Foley catheter (Foley catheter) inserted into the nasal puncta. Motion artefact was present in all the scans because of the standing sedation method employed but did not significantly affect the diagnostic quality of the images. The examination revealed mild soft tissue swelling associated with underlying marked bilateral irregular periosteal proliferation of the caudal aspects of the nasal bones, lacrimal bone, rostral aspect of the frontal bone and the nasofrontal-, nasolacrimal- frontolacrimal-, lacrimozygomatic and lacrimomaxillary suture areas (Figure 5a, b, c, d and e). The most severe proliferation affected the lacrimal bone sutures. The contrast medium could be visualised from the nasal puncta bilaterally up to the level of Triadan 110 and 210. There was obvious narrowing and stenosis of the lumen of the right nasolacrimal duct at the level of the most severe bony changes (lacrimomaxillary suture line), with complete absence of the contrast medium at this site, but it reappeared proximally up to the lacrimal puncta. Similar findings were present on the left side, with the obstruction being present more proximally at the level of the lacrimal puncta, but contrast was found within the lacrimal lake. These findings confirmed the partial bilateral nasolacrimal obstruction.

Minimally displaced depression fractures of the right nasal bone, the right maxillary bone and right frontal bone were demonstrated, despite the presence of motion artefact. Surgical intervention was not deemed necessary based on the mild displacement, good stability of the fracture and callus formation.

At the completion of the CT examination and with confirmation of the patency of both nasolacrimal ducts, retrograde flushing of both ducts was performed successfully, although significant pressure had to be applied to the right nasolacrimal duct. The mare was discharged with instructions of stable confinement for 6 weeks, and a therapeutic regimen of ocular chloramphenicol (25 mg/kg bwt Chlorcol, Adcock Ingram, Midrand, South Africa) twice daily for 5 days and oral meloxicam (0.6 mg/kg bwt Metacam, Boehringer Ingelheim, Johannesburg, South Africa) once daily for 14 days.

A re-examination 6 weeks post-presentation and 12 weeks after the initial traumatic episode revealed that the mare had normal clinical parameters and mentation. A firm area of swelling was evident extending between the medial canthi of both eyes. Palpation of this swelling revealed a moderate pain response, but this was markedly reduced compared to 6 weeks previously. There was no evidence of epiphora or conjunctivitis, and the owners reported the mare to be showing significantly less resentment to general palpation of the facial region.

A second radiographic evaluation was performed under a standing sedation protocol, which revealed no significant differences compared to the initial radiographs.

The owners were requested to maintain the mare in stable confinement and to commence hand walking and trot work on the lunge over the next 6 weeks. A telephone update at this point confirmed that the mare had made excellent progress, and small paddock turnout was commenced.

**FIGURE 6:**
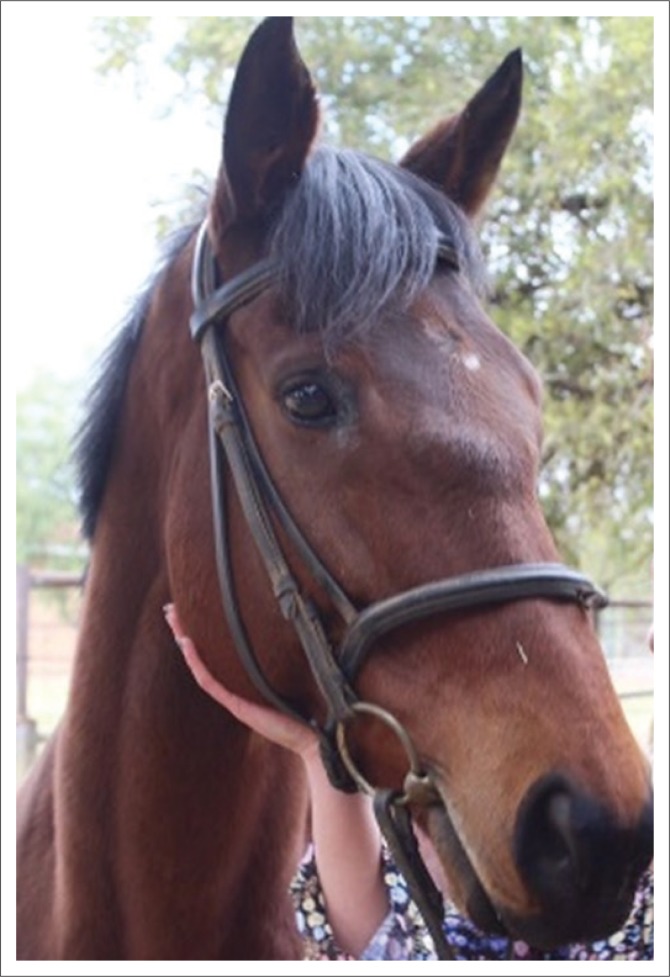
Eight months after the initial traumatic episode. A smaller area of swelling is still evident on the nasofrontal suture line.

Eight months after the initial injury, the mare had returned to her normal routine, although a firm swelling was still present at the nasofrontal suture line area. The lacrimal and maxillary bones appeared normal (Figure 6). The mare showed no change in demeanour, no pain on palpation of the nasal, facial or lacrimal bones, no resentment to having a bridle with noseband placed and no decrease in appetite. The owners were satisfied with the outcome and had commenced normal ridden work with the mare.

## Discussion

Local facial trauma has been suggested as the likely aetiology of the inflammatory response seen in suture line exostosis (Carslake 2009; Dixon 1991, 2014; Gibbs & Lane 1987). This aetiology has also been suggested as the reason for the often-rapid development of these lesions (Dixon 1991; Tremaine & Freeman 2006).

Although, like the mare in this case, some affected horses have a definite history of a single episode of head trauma, present with acute-onset epistaxis or have a history of recent facial bone surgery, the majority of cases of suture line exostosis do not. Two studies reporting a series of suture exostosis cases revealed 0/16 cases (Gibbs & Lane 1987) and 5/26 cases (Tremaine & Dixon 2001b) to have had a known history of head trauma. It has been suggested that facial trauma may occur unseen in the field, thus complicating identification of this aetiology (Carslake 2009). Additionally, some authors have challenged this theory, highlighting the apparent bilateral symmetry of these lesions (Klein et al. 2014). It has been shown in other species that masticatory forces could contribute to the development or persistence of suture line exostosis, with large tensile and compressive forces demonstrated across suture lines during mastication in miniature swine (Rafferty & Herring 1999). The role of masticatory forces in the development of suture line exostosis in horses has subsequently been suggested (Bonilla et al. 2015; Carslake 2009; Dixon 2014). Inflammation of the nasofrontal and nasolacrimal suture lines have also been reported following frontal sinus trephination or sinonasal surgery (Manso-Diaz & Taeymans 2012; Tremaine & Dixon 2001a; Woodford & Lane 2006) and in conjunction with additional pathology such as a nasal septum chondrosarcoma (Bonilla et al. 2015).

Clinical signs of suture line exostosis of the nasolacrimal, maxillolacrimal and frontolacrimal suture lines commonly include bilateral diffuse swellings situated rostrally to the medial canthus of the eye with or without accompanying epiphora (Carslake 2009; Dixon 1991; Klein et al. 2014; Manso-Diaz & Taeymans 2012; Tremaine & Dixon 2001a). These swellings have consistently been reported as non-painful (Bonilla et al. 2015; Carslake 2009; Klein et al. 2014; Manso-Diaz & Taeymans 2012), which made the severely painful swellings in this case unusual. The severity of the pain evident in this case made us consider the possibility of fractures of the facial bones being present. The mare in this report initially had three discrete swellings that then developed into one horizontal swelling corresponding to the nasofrontal suture line. Occasionally exudation through the overlying skin can occur with suture line exostosis, but this is unusual (Dixon 2014) and was not present in this case. Epiphora because of suture line exostoses is normally due to swelling and discharge obstructing the nasolacrimal puncta and duct, thus reducing lacrimal drainage (Carslake 2009; Manso-Diaz & Taeymans 2012). In this case, the obstruction of both nasolacrimal ducts was shown to be the cause of the epiphora with a negative Jones test on both sides and resistance encountered during flushing of each duct.

Radiographic evaluation is the most common method of diagnosing suture exostosis (Butler et al. 2009; Dixon 1991; Gibbs & Lane 1987; Tremaine & Dixon 2001a; Wyn-Jones 1985). Characteristic features of early, active suture exostoses on lateral radiographs are a central radiolucent area between two areas of sclerotic bone, with thickening of periosteal new bone towards the suture line (Butler et al. 2009; Dixon 1991; Gibbs & Lane 1987; Tremaine & Dixon 2001a; Wyn-Jones 1985). The radiographic evaluation in this case was useful to make a tentative diagnosis of the condition and the presence of facial fractures. Computed tomography has been shown to be superior to radiography for the identification of complex anatomic structures and traumatic injuries of the maxillofacial region in dogs and cats (Bar-Am et al. 2008) and horses (Manso-Díaz et al. 2015). In a comparison of the imaging modalities available to assess suture line exostosis, CT and magnetic resonance imaging (MRI) examinations have been shown to provide superior resolution and precise assessment of the bony structures and periosteal changes involved in suture line exostosis (Arencibia et al. 2000; Bonilla et al. 2015; Manso-Diaz & Taeymans 2012; Nykamp, Scrivani & Pease 2004). These techniques also avoid superimposition of structures and provide serial cross-sectional images of the nasolacrimal duct (Arencibia et al. 2000; Manso-Diaz & Taeymans 2012; Nykamp et al. 2004). The use of contrast medium to enhance CT examinations and outline the nasolacrimal ducts is recommended and provides a mechanism to differentiate between obstruction of the duct by bony proliferation and intra-luminal inflammatory products (Manso-Diaz & Taeymans 2012; Nykamp et al. 2004). Although the CT images were diagnostic in this case, motion artefacts were evident in all scans. This may be related to the use of detomidine hydrochloride and butorphanol tartrate in the sedation protocol. A different sedation protocol using alternative agents, for instance, romifidine hydrochloride, may have decreased the motion artefacts seen.

In this case, the resolution afforded by CT with the use of dacryocystorhinography confirmed partial obstruction of both nasolacrimal ducts and allowed the severity and location of the nasolacrimal duct obstructions to be determined.

Dacryocystorhinography has been frequently performed under standing sedation (Carslake 2009; Cassotis & Schiffman 2006; Ramzan & Payne 2005; Schumacher, Dean & Welch 1992), and the development of standing CT examinations with dacryocystorhinography avoids the associated risks of general anaesthesia. Additionally, the majority of suture line exostosis cases do not require surgical intervention, and many potential surgical treatments can also now be achieved under a standing sedation protocol (Brink & Schumacher 2016; Robinson et al. 2016).

Dacryocystorhinography is a moderately technically demanding procedure and can be irritating for the horse because of the pressure within the duct (Carslake 2009). It has been reported that excessive pressure within the duct can cause the duct to rupture, and the contrast agent can be an irritant to surrounding tissues (Butler et al. 2009). In our opinion, a CT examination evaluating the patency of the nasolacrimal duct is required before significant pressure is applied to flush a blocked lumen and duct to remove intra-luminal inflammatory secretions.

After accurate identification, craniofacial suture line exostoses are normally treated successfully with stable confinement and anti-inflammatory treatment (Bonilla et al. 2015; Carslake 2009; Dixon 1991; Gibbs & Lane 1987; Tremaine & Dixon 2001b). Acute soft tissue inflammation associated with exostosis in other regions of the horse, for instance, the small metacarpal bones in the equine appendicular skeleton, has been managed with local and systemic anti-inflammatory drugs, hypothermia and radiation therapy (Bertone 2002; Carslake 2009; Theon 2003). These treatment modalities have been suggested in the management of early craniofacial suture line exostosis (Carslake 2009).

The significant improvement in this case after 8 months has been previously recorded in studies assessing the long-term outcome of these cases (Carslake 2009; Dixon 2014; Tremaine & Dixon 2001b). Nasofrontal suture exostosis can take 12–18 months to completely regress and regain a normal contour (Dixon 2014). Nasolacrimal suture line exostosis has been reported to significantly improve and the accompanying epiphora to resolve within a few months, as in this case (Dixon 2014).

Reassessment and surgical excision or stabilisation has been recommended in cases with persistent epiphora, large lesions unresponsive to conservative therapy, or in cases where the cosmetic appearance and epiphora are unacceptable to the owners (Bonilla et al. 2015; Carslake 2009; Klein et al. 2014; Manso-Diaz & Taeymans 2012). Reported treatments include excision of the periosteal reaction, injection of the suture line with platelet rich plasma, suturing of the nasofrontal periosteum, stabilisation of the nasal and frontal bones with a surgical implant and canaliculorhinostomy (Carslake 2009; Klein et al. 2014; Manso-Diaz & Taeymans 2012; McIlnay, Miller & Dugan 2001; Wilson & Levine 1991). The importance of CT imaging in these cases has been highlighted to allow for surgical planning and the precise assessment of related bony structures (Dixon 2014; Klein et al. 2014; Manso-Diaz & Taeymans 2012), especially in more complex and extensive cases such as the case described.

It is the opinion of the authors that CT examination with positive contrast dacryocystorhinography is a very useful technique for the diagnosis of suture line exostosis and evaluation of the patency of the nasolacrimal ducts when the nasofrontal or lacrimomaxillary suture lines are affected and epiphora is present. There is minimal risk to the horse as both diagnostic techniques can be performed in the standing, sedated horse. However, a sliding gantry design CT is required for standing equine CT to be performed. Further studies utilising CT will be of benefit to assess the complexities and relationships of suture line exostosis, as well as to define the relationship between fractures and exostosis.
